# Analysis of potential genes and pathways associated with the colorectal normal mucosa–adenoma–carcinoma sequence

**DOI:** 10.1002/cam4.1484

**Published:** 2018-04-16

**Authors:** Zhuoxuan Wu, Zhen Liu, Weiting Ge, Jiawei Shou, Liangkun You, Hongming Pan, Weidong Han

**Affiliations:** ^1^ Department of Medical Oncology Sir Run Run Shaw Hospital College of Medicine Zhejiang University Hangzhou, Zhejiang China; ^2^ Cancer Institute The Second Affiliated Hospital College of Medicine Zhejiang University Hangzhou, Zhejiang China

**Keywords:** Colorectal normal mucosa–adenoma–carcinoma sequence, differentially expressed genes, functional analysis, microarray analysis, prognosis

## Abstract

This study aimed to identify differentially expressed genes (DEGs) related to the colorectal normal mucosa–adenoma–carcinoma sequence using bioinformatics analysis. Raw data files were downloaded from Gene Expression Omnibus (GEO) and underwent quality assessment and preprocessing. DEGs were analyzed by the limma package in R software (R version 3.3.2). Gene Ontology (GO) enrichment analysis and Kyoto Encyclopedia of Genes and Genomes (KEGG) pathway analysis were performed with the DAVID online tool. In a comparison of colorectal adenoma (*n* = 20) and colorectal cancer (CRC) stage I (*n* = 31), II (*n* = 38), III (*n* = 45), and IV (*n* = 62) with normal colorectal mucosa (*n* = 19), we identified 336 common DEGs. Among them, seven DEGs were associated with patient prognosis. Five (*HEPACAM2, ITLN1, LGALS2, MUC12,* and *NXPE1*) of the seven genes presented a sequentially descending trend in expression with tumor progression. In contrast, *TIMP1* showed a sequentially ascending trend. *GCG* was constantly downregulated compared with the gene expression level in normal mucosa. The significantly enriched GO terms included extracellular region, extracellular space, protein binding, and carbohydrate binding. The KEGG categories included HIF‐1 signaling pathway, insulin secretion, and glucagon signaling pathway. We discovered seven DEGs in the normal colorectal mucosa–adenoma–carcinoma sequence that was associated with CRC patient prognosis. Monitoring changes in these gene expression levels may be a strategy to assess disease progression, evaluate treatment efficacy, and predict prognosis.

## Introduction

Colorectal cancer (CRC) is the third leading cause of cancer and cancer‐related death in patients with cancer worldwide, accounting for more than 134,000 estimated new cases and 49,000 estimated deaths in 2016 1. The five‐year survival rate of CRC is approximately 65% in high‐income countries but is <50% in low‐income countries 2, 3, 4. From 2000 to 2014, the mortality of CRC decreased by 18% in individuals aged ≥50 years due to the extensive use of traditional screening methods, including flexible colonoscopy, barium enema X‐ray, and fecal blood testing 5. However, these tests have non‐negligible shortcomings, including bleeding, perforation, and acute diverticulitis, as well as a variable sensitivity ranging from 37% to 80% 6, 7. Therefore, sensitive and specific biomarkers are urgently needed to improve the rate of early diagnosis, to help manage individual therapy and to predict the prognosis of patients in different stages of the disease.

Most CRC cases develop slowly through the normal mucosa–adenoma–carcinoma sequence over several years 8. During this multistep process of colorectal tumorigenesis, many factors play important roles, including old age, smoking, alcohol, a high‐fat diet, and lack of physical exercise 9. In recent decades, multiple genes and signaling pathways have been shown to participate in the initiation and development of CRC. Kinzler and Fearon et al. reported that *APC* inactivation was an early event in more than 70% of colorectal adenomas and carcinomas 10, 11. *KRAS* and *TP53* mutations participated in the adenoma–carcinoma sequence 11. Liu et al. 12 found that low miR‐126 and high CXCR4 protein expression were associated with poor prognosis in colorectal cancer. Tsukamoto et al. 13 reported that overexpression of osteoprotegerin in human colorectal cancer might be a predictive biomarker of CRC recurrence and a potential target for individual treatment of this disease. Dynamic changes of genes in different stages have important roles in the occurrence and development of CRC, as well as the treatment and prognosis of this disease 14, 15, 16, 17. These differentially expressed genes (DEGs) may show changes that correspond to their functions in the different stages of CRC, which lead to different survival outcomes 18. Stage at diagnosis is an important prognostic factor for patients with CRC. Siegel et al. 2 found that the five‐year survival rate of patients diagnosed with CRC ranges from 90.1% in stage I to 11.7% in stage IV. Thus, it is important to identify DEGs during the normal mucosa–adenoma–carcinoma sequence, which will help elucidate the molecular mechanisms involved in the occurrence and development of CRC, provide potential biomarkers for diagnosis at the early stage, and suggest potential targets for individual therapy.

Bioinformatics is a newly emerging scientific field that combines biology, mathematics, and information technology, making it possible to analyze large and increasingly complex molecular datasets. Microarray assays can acquire expression information on thousands of genes simultaneously and explore the genomic alterations associated with the progress of colorectal initiation and development 19. Extensive genetic information is available online due to the development of public cancer databases, such as The Cancer Genome Atlas (TCGA), Oncomine, Gene Expression Omnibus (GEO), and others, which are repositories for microarray data retrieval and deposit. Online datasets can help enlarge the sample size and increase the statistical power. For example, Fu et al. 20 identified 72 miRNA–mRNA pairs along with 22 dysregulated miRNAs and their 58 target mRNAs that were involved in CRC tumorigenesis by a combination of expression profiling and bioinformatics analysis. Robles et al. 21 found that the CRC that developed in patients with IBD had different genetic characteristics from sporadic CRC with whole‐exome sequencing analysis, providing possible genetic biomarkers for diagnosis and treatment of patients with IBD and CRC.

In our study, we aimed to identify DEGs related to the colorectal normal mucosa–adenoma–carcinoma sequence. Original data were downloaded from GEO and analyzed with R software (R version 3.3.2). Gene expression levels in colorectal adenoma and CRC stage I, II, III, and IV were compared with those in normal colorectal mucosa. We eventually identified seven potential DEGs related to CRC patient prognosis and explored their function by performing Gene Ontology (GO) analysis and Kyoto Encyclopedia of Genes and Genomes (KEGG) pathway analysis.

## Materials and Methods

### Screening microarray data

The GEO database was systematically searched without language, race, region, and time restrictions (up to 7 January 2017). The advanced search strategy insured the comprehensiveness of the search results (see Table S1 for search strategy details). These inclusion criteria were as follows: (1) total RNA was extracted from frozen colorectal tissue sections; (2) datasets had the original gene expression data files. RNA extracted from frozen tissues shows little degradation. This was the basis for the qualified microarray. In addition, the original gene expression data files could realistically indicate the microarray quality. Evaluating chip quality and rejecting unqualified chip data insured the accuracy of the subsequent analysis. The exclusion criteria were as follows: (1) the genome‐wide gene expression profile was not generated by Affymetrix Human Genome U133 Plus 2.0 Array; (2) No disease staging information was present; (3) Frozen tissue sections came from patients who might have received antitumor treatments previously. At present, there is no readymade dataset containing normal colorectal mucosa, adenoma, and carcinoma with all four stages in one dataset. However, there were several datasets containing either colorectal adenoma or carcinoma at different stages. The integration of these different datasets combined the normal colorectal mucosa, adenoma, and carcinoma at all four stages together, making it possible to analyze genetic changes in the progression of colorectal cancer. Different datasets had heterogeneity, but the heterogeneity was much smaller if the datasets were generated from one platform. The Affymetrix Human Genome U133 Plus 2.0 Array was selected because this platform generated the most available datasets for further analysis. At the same time, it was necessary to exclude the influence of the therapeutic factors on the gene expression level. Thus, patients who previously received antitumor treatments were excluded.

### Evaluation of microarray quality

The selected gene expression data were downloaded from the GEO database. These raw data files were from the Affymetrix platform, which could be analyzed by the affy package 22 in R software (R version 3.3.2). Before data preprocessing, all the microarrays were evaluated for quality by quality control (QC), relative logarithmic expression (RLE), normalized unscaled standard errors (NUSE), and RNA degradation curve 23, 24. QC is an overall assessment of the microarray quality, which primarily consists of the present percentage, background noise, scale factor, GAPDH 3'/5' ratio, and actin 3'/5' ratio. RLE and NUSE can both evaluate the consistency of the data, while NUSE is more sensitive. The RNA degradation curve plays an important role in evaluating the degradation of the microarray. Through quality assessment, poor quality data were removed.

### Processing of microarray data

Data preprocessing was performed with a standard robust multiarray average (RMA) method, including background correction, normalization, and logarithmic conversion 25. The raw data were converted to probe‐level data after the RMA algorithm and were then transformed to gene symbols in R software 26. The gene expression levels were the mean of the probes when multiple probes corresponded to one gene symbol. The batch effect could be due to different experimental times, methods, experimenters, datasets, platforms, and many other unpredictable factors, which might affect the accuracy of the data analysis. However, the datasets generated from one platform have a much smaller batch effect. In addition, the batch effect was evaluated by the expression level of housekeeping genes in each dataset to judge whether batch effects have a significant impact on our conclusions. The DEGs were identified by the limma package in R software 27. Only genes with |log_2_FC| >1 (FC: fold change) and an adjusted *P*‐value <0.05 were considered DEGs. Then, all DEGs underwent prognostic analysis with the survival information from TCGA. TCGA database had no separate colorectal adenoma data, and thus, this information was only used to validate the DEGs in colorectal cancer with four stages with RNA‐seq data.

### Functional and pathway enrichment analysis

GO annotates and classifies genes based on three categories, including biology process, molecular function, and cellular component 28, 29. KEGG pathway interprets pathway maps of molecular interactions, reactions, and relation networks 30. In our analysis, the DAVID online tool was used to perform GO enrichment and KEGG pathway analysis of the identified DEGs and many other related background DEGs with threshold *P*‐values <0.05.

## Results

### Basic characteristics of the microarray data

**Figure 1:**
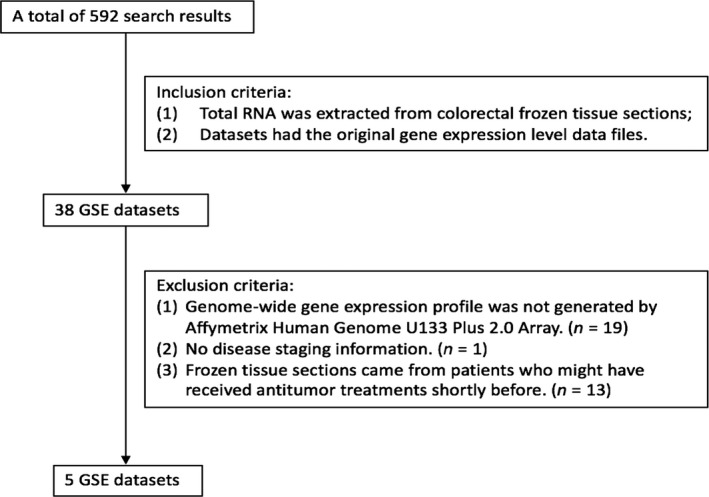
Flowchart of selecting eligible datasets.

A total of 592 search results were obtained by our search strategy (See Table S1 for search strategy details). Thirty‐eight datasets met the two inclusion criteria. Their RNAs were all extracted from frozen colorectal tissue sections. In addition, these 38 datasets had the original gene expression data files. Based on the exclusion criteria, 19 datasets were excluded because the Affymetrix Human Genome U133 Plus 2.0 Array was not used. One dataset was excluded because it had no staging information. Thirteen datasets with patients who might have received antitumor treatments shortly before were also excluded. Thus, only five datasets (GSE4183, GSE14333, GSE39582, GSE8671, and GSE10714) were finally eligible for analysis (Fig. 1, Table S2).

These five datasets had a total of 971 raw data files, which were from the Affymetrix platform. According to the staging information, data were divided into six groups: normal mucosa, adenoma, and CRC stage I, II, III, and IV. Quality assessment was performed for all these raw data files by QC, RLE, NUSE, and the RNA degradation curve (Fig. S1). At the same time, the consistency of the data volume of each group and the microarray quality were taken into account, and 215 data profiles were finally included in the analysis (Table 1). In addition, the batch effect was evaluated with the expression level of GAPDH across the different datasets, and heterogeneity was not significant (Fig. S2).

**Table 1:** GSE datasets included in our study

Sample stage	Quantity	GSE datasets
Colorectal normal mucosa	19	GSE4183 + GSE8671
Adenoma	20	GSE4183 + GSE8671
CRC stage 1	31	GSE14333 + GSE39582
CRC stage 2	38	GSE14333
CRC stage 3	45	GSE14333
CRC stage 4	62	GSE14333 + GSE39582

CRC, colorectal cancer; GEO, Gene Expression Omnibus; GSE, GEO series.

### Identification of DEGs and prognosis analysis

**Figure 2:**
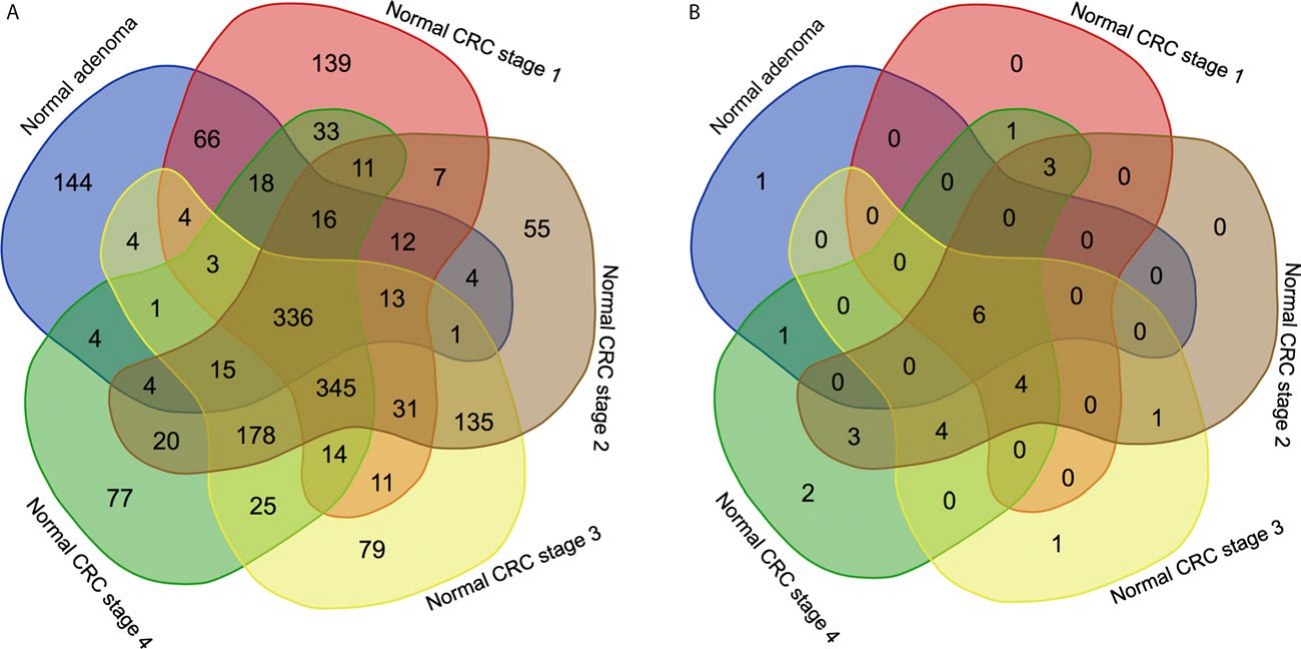
Venn diagram for (A) the 336 common DEGs with threshold of |log_2_ FC| >1 and an adjusted *P*‐value <0.05 and for (B) the six common DEGs with threshold of |log_2_ FC| >4 and an adjusted *P*‐value <0.05 extracted from normal mucosa–adenoma, normal mucosa–CRC stage I, normal mucosa–CRC stage II, normal mucosa–CRC stage III, and normal mucosa–CRC stage IV. FC, fold change.

**Figure 3:**
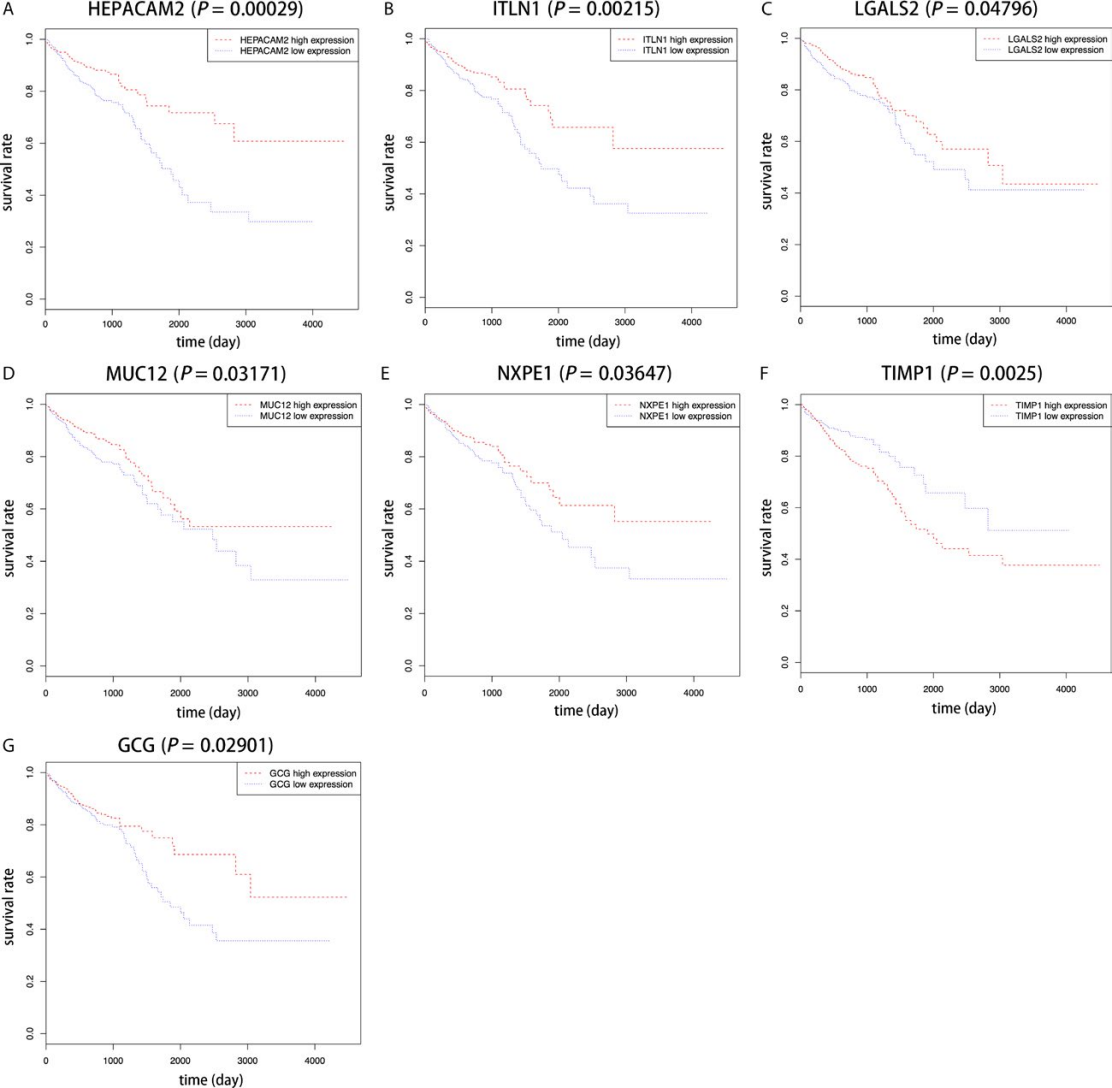
Survival curve of (A) HEPACAM2, (B) ITLN1, (C) LGALS2, (D) MUC12, (E) NXPE1, (F) TIMP1, and (G) GCG.

The gene expression levels in the colorectal adenoma and CRC stage I, II, III, and IV were compared to those in normal colorectal mucosa. The five comparison groups were normal mucosa–adenoma, normal mucosa–CRC stage I, normal mucosa–CRC stage II, normal mucosa–CRC stage III, and normal mucosa–CRC stage IV. With a threshold of |log_2_FC| >1 and an adjusted *P*‐value <0.05, 645 DEGs were identified between the normal mucosa and the adenoma. In addition, there were 1059, 1183, 1195, and 1100 DEGs corresponding to normal mucosa–CRC stage I, normal mucosa–CRC stage II, normal mucosa–CRC stage III, and normal mucosa–CRC stage IV, respectively. Then, 336 *common* DEGs were extracted from these five comparison groups (Fig. 2A). Among these 336 *common* DEGs, 87 genes were identified with an ascending or descending trend through the normal mucosa–adenoma–carcinoma sequence (Table S3). Using the survival information of TCGA, we eventually selected six DEGs related to patient prognosis. They were *HEPACAM2, ITLN1, LGALS2, MUC12, NXPE1*, and *TIMP1* (Fig. 3A–F, Table 2). Five of the six genes presented a descending trend in expression with tumor progression, while one gene presented an ascending trend.

**Table 2:** Six genes presenting sequentially expression level changes through normal colorectal mucosa–adenoma–carcinoma sequence

Genes	Normal adenoma |FC|	Normal stage 1 |FC|	Normal stage 2 |FC|	Normal stage 3 |FC|	Normal stage 4 |FC|	Five‐year survival rate (*P*‐value)
HEPACAM2	−1.21761	−3.85613	−4.0706	−4.06757	−4.57233	0.00029
ITLN1	−2.18651	−4.00026	−4.54605	−4.00429	−4.82109	0.00215
LGALS2	−1.86415	−2.7824	−3.50298	−3.01946	−3.08688	0.04796
MUC12	−1.08214	−2.12846	−2.39063	−2.5668	−2.54186	0.03171
NXPE1	−1.00081	−2.57598	−2.93155	−2.77359	−3.2236	0.03647
TIMP1	1.561751	1.690323	2.036284	2.050785	2.217349	0.0025

FC, fold change.

We also obtained another six common DEGs from these five comparison groups with a threshold of |log_2_FC| >4 and an adjusted *P*‐value <0.05. They were *AQP8, CLCA4, CLDN8, GCG, GUCA2A*, and *MS4A12* (Fig. 2B, Table 3). These genes were all downregulated compared with the gene expression levels in normal mucosa. Among them, only *GCG* was considered related to patient prognosis (Fig. 3G, Table 3).

**Table 3:** Six genes maintained continuous downregulated compared with gene expression level in normal colorectal mucosa

Genes	Normal adenoma |FC|	Normal stage 1 |FC|	Normal stage 2 |FC|	Normal stage 3 |FC|	Normal stage 4 |FC|	Five‐year survival rate (*P*‐value)
AQP8	−5.08128	−5.59115	−5.85151	−5.60485	−6.51036	0.13895
CLCA4	−4.0168	−5.75406	−5.75458	−5.46981	−6.55938	0.16333
CLDN8	−4.71209	−5.19946	−5.58836	−5.06006	−5.65663	0.9914
GCG	−5.4652	−5.40381	−5.95615	−5.65407	−5.70174	0.02901
GUCA2A	−4.11537	−4.40829	−4.32434	−4.24492	−4.73345	0.20505
MS4A12	−4.48105	−5.56404	−5.52912	−5.06747	−6.16997	0.74113

FC, fold change.

A total of seven DEGs from the GEO database associated with prognosis were obtained by bioinformatics analysis. They were *HEPACAM2, ITLN1, LGALS2, MUC12, NXPE1, TIMP1*, and *GCG*. The expression patterns of these seven DEGs were also confirmed by 672 RNA‐seq data from TCGA. Their expression levels all presented the same trend as that in the GEO database except *MUC12* (Table 4). However, *MUC12* was also downregulated in colorectal cancer tissues compared with normal colorectal mucosa. The expression of these seven DEGs in colorectal adenomas could not be verified because TCGA database had no separate adenoma data.

**Table 4:** The expression pattern of seven DEGs on the TCGA

Genes	Normal stage 1 |FC|	Normal stage 2 |FC|	Normal stage 3 |FC|	Normal stage 4 |FC|
HEPACAM2	−4.6630	−4.4554	−4.8929	−5.5554
ITLN1	−5.3944	−5.3083	−5.4875	−6.0155
LGALS2	−3.5660	−3.8652	−3.5149	−3.9496
MUC12	−2.7603	−2.8897	−2.4149	−2.5861
NXPE1	−3.6871	−3.7573	−4.0759	−4.0087
TIMP1	1.1477	1.4022	1.5606	1.4898
GCG	−7.1766	−7.2416	−6.8744	−6.0155

DEGs, differentially expressed genes.

*P* < 0.05.

### GO term enrichment analysis

Using a threshold of |log_2_FC| >1 and an adjusted *P*‐value <0.05, we identified 645, 1059, 1183, 1195, and 1100 DEGs in the comparisons of normal mucosa–colorectal adenoma, normal mucosa–CRC stage I, normal mucosa–CRC stage II, normal mucosa–CRC stage III, and normal mucosa–CRC stage IV, respectively. Combining these genes together, we obtained a total of 1805 background DEGs in a union including the seven identified DEGs.

These 1805 DEGs were uploaded to the DAVID online tool to perform GO analysis and KEGG pathway analysis to explore the possible biological functions and signaling pathways of the DEGS. The results of the seven identified DEGs were extracted separately. In the biology process GO category, the functional enrichment of the seven DEGs was scattered so that no common biology process was found among these seven DEGs. In the cellular component GO category, *TIMP1, GCG*, and *NXPE1* were related to extracellular region, and *TIMP1* and *GCG* were related to extracellular space. In the molecular function GO category, *HEPACAM2, LGALS2, TIMP1,* and *GCG* were associated with protein binding, and *ITLN1* and *LGALS2* were associated with carbohydrate binding. Additionally, these DEGs had some other specific classifications (Table 5).

**Table 5:** GO term enrichment analysis of seven DEGs

Genes	Species	Biology process	Cellular component	Molecular function
HEPACAM2	Homo sapiens	GO:0007067~mitotic nuclear division, GO:0051301~cell division,	GO:0005819~spindle, GO:0030496~midbody,	GO:0005515~protein binding,
ITLN1	Homo sapiens	GO:0001934~positive regulation of protein phosphorylation,	GO:0031225~anchored component of membrane, GO:0031526~brush border membrane, GO:0070062~extracellular exosome,	GO:0030246~carbohydrate binding,
LGALS2	Homo sapiens			GO:0005515~protein binding, GO:0030246~carbohydrate binding,
MUC12	Homo sapiens	GO:0001558~regulation of cell growth, GO:0016266~O‐glycan processing,	GO:0005796~Golgi lumen, GO:0005887~integral component of plasma membrane,	
NXPE1	Homo sapiens		GO:0005576~extracellular region,	
TIMP1	Homo sapiens	GO:0002576~platelet degranulation, GO:0009725~response to hormone, GO:0022617~extracellular matrix disassembly, GO:0034097~response to cytokine, GO:0042060~wound healing, GO:0043066~negative regulation of apoptotic process, GO:0043434~response to peptide hormone, GO:0051216~cartilage development,	GO:0005576~extracellular region, GO:0005578~proteinaceous extracellular matrix, GO:0005581~collagen trimer, GO:0005604~basement membrane, GO:0005615~extracellular space, GO:0031093~platelet alpha granule lumen, GO:0070062~extracellular exosome,	GO:0002020~protease binding, GO:0005125~cytokine activity, GO:0005515~protein binding, GO:0008083~growth factor activity,
GCG	Homo sapiens	GO:0008283~cell proliferation, GO:0010800~positive regulation of peptidyl‐threonine phosphorylation, GO:0043066~negative regulation of apoptotic process, GO:0070374~positive regulation of ERK1 and ERK2 cascade,	GO:0005576~extracellular region, GO:0005615~extracellular space, GO:0005788~endoplasmic reticulum lumen, GO:0005886~plasma membrane,	GO:0005102~receptor binding, GO:0005179~hormone activity, GO:0005515~protein binding,

DEGs, differentially expressed genes; GO, Gene Ontology analysis.

### KEGG pathway analysis

The KEGG pathway enrichment analysis indicated that *TIMP1* might participate in the HIF‐1 signaling pathway, and *GCG* might play a role in the insulin secretion and glucagon signaling pathway (Table 6).

**Table 6:** KEGG pathway analysis of seven DEGs

Genes	Species	KEGG pathway
HEPACAM2	Homo sapiens	/
ITLN1	Homo sapiens	/
LGALS2	Homo sapiens	/
MUC12	Homo sapiens	/
NXPE1	Homo sapiens	/
TIMP1	Homo sapiens	hsa04066: HIF‐1 signaling pathway,
GCG	Homo sapiens	hsa04911: Insulin secretion, hsa04922: Glucagon signaling pathway,

DEGs, differentially expressed genes; KEGG, Kyoto Encyclopedia of Genes and Genomes Pathway.

## Discussion

Sequential changes of gene expression in different stages play essential roles in the colorectal normal mucosa–adenoma–carcinoma sequence 31. Many studies have confirmed that DEGs participated in the progress of CRC. Heijink et al. 32 reported that caspase‐8 and cellular flice‐like inhibitory protein (cFLIP) expression induced colorectal carcinogenesis independently in sporadic and hereditary nonpolyposis colorectal cancer (HNPCC)‐associated adenomas and carcinomas. Galamb et al. 24 showed that downregulated amnionless homolog (AMN) and prostaglandin‐D2 receptor (PTGDR) and upregulated osteopontin and osteonectin were potential biomarkers of colorectal carcinogenesis and disease progression. However, most studies only focused on the colorectal carcinogenesis process, from normal colorectal mucosa to CRC 33, 34. Many other studies have examined the macro classification, such as from normal colorectal mucosa to adenoma and then to CRC with all stages mixed 35, 36. Comparatively speaking, our study had a more detailed grouping because there were five comparison groups in total. The gene expression levels in the colorectal adenoma and CRC stage I, II, III, and IV were compared with those of the normal colorectal mucosa. This has not been performed previously in the current literature. By analyzing public gene data from GEO and TCGA in R software (R version 3.3.2), we eventually identified seven DEGs potentially related to CRC patient prognosis and explored their function by performing GO analysis and KEGG pathway analysis. These findings may help elucidate the molecular mechanisms involved in the initiation and development of CRC, provide potential biomarkers of early diagnosis, help manage potential targets in individual therapy, and predict the prognosis of patients at different stages of the disease.

In our study, we downloaded GSE4183, GSE14333, GSE39582, GSE8671, and GSE10714 from the public database GEO, which is an international public repository for microarray data retrieval and deposit. These data were submitted by the investigators, and there was a lack of quality review and evaluation 37. Thus, evaluating the quality of microarray assays is important. QC is an overall assessment of the microarray quality, which primarily consists of present percentage, background noise, scale factor, GAPDH 3'/5' ratio, and actin 3'/5' ratio 24. RLE and NUSE can both evaluate the consistency of the data, while NUSE is more sensitive. The RNA degradation curve plays an important role in evaluating the degradation of the microarray 24. For RLE, the box chart center of each data profile in the high‐quality dataset should be close to the position of the ordinate 0. For NUSE, it should be close to 1. If the slope of the RNA degradation curve is close to 0, it indicates that the degradation of the microarray is serious, and these data should be removed. The raw data must go through these quality evaluations, and only qualified data can be entered into the next data processing step to insure the reliability of the subsequent analysis 38.

We obtained seven DEGs of interest, which were *HEPACAM2, ITLN1, LGALS2, MUC12, NXPE1, TIMP1*, and *GCG*, and all were associated with patient prognosis. *HEPACAM2, ITLN1, LGALS2, MUC12*, and *NXPE1* presented a sequentially descending trend in expression with tumor progression. In contrast, *TIMP1* presented a sequentially ascending trend. Furthermore, *GCG* showed constant downregulation compared with the gene expression level in normal mucosa. Among these seven DEGs, *TIMP1* and *GCG* have been studied extensively. *TIMP1* is a member of the tissue inhibitors of metalloproteinase (*TIMP*) family that regulates matrix metalloproteinases (MMPs) and disintegrin metalloproteinases 39. Recent studies reported that the dysregulated activity of *TIMP1* was associated with cancer progression 40. Increased expression of *TIMP1* was shown to predict worse prognosis of laryngeal carcinoma 41 and melanoma 42. Many studies have reported the *TIMP1* was upregulated in both early and advanced CRC 43, 44, and it possibly acted as a prognostic biomarker involved in liver metastasis of CRC 45, 46. In our study, we found that *TIMP1* presented a sequentially ascending trend through the normal colorectal mucosa–adenoma–carcinoma sequence, and the upregulation of *TIMP1* indicated a poor survival prognosis, consistent with previous studies. *GCG* is involved in the regulation of incretin synthesis, secretion, inactivation, and RET signaling. Diseases related to *GCG* are diabetes 47 and other metabolic diseases 48. Drucker 49 reported that the protein encoded by *GCG* was a preproprotein, which could be cleaved into four mature peptides and regulated cell proliferation, differentiation, and apoptosis. However, few studies have focused on the role of *GCG* in CRC progression. We discovered that *GCG* expression was downregulated in both adenomas and carcinomas, which was also confirmed by Spisak et al. 50.

There are few studies about *HEPACAM2, ITLN1, LGALS2, MUC12*, and *NXPE1*, even rare in CRC. These five genes all presented a sequentially descending trend in expression through the normal colorectal mucosa–adenoma–carcinoma sequence. *HEPACAM2* is a member of the immunoglobulin family of adhesion genes. The clinical importance of *HEPACAM2* in CRC remains unclear. *ITLN1* encodes intelectin‐1, which functions as a receptor for both bacterial arabinogalactans and lactoferrin. Li et al. 51 noted that intelectin‐1 suppressed tumor progression and was associated with improved survival in gastric cancer. However, there is no research exploring the function of *ITLN1* in CRC. *LGALS2* encodes galectin‐2, which participates in non‐small‐cell lung cancer 52, coronary heart disease 53, and ischemic stroke 54 instead of CRC. *MUC12* is a member of mucin family. Matsuyama et al. 55 reported that *MUC12* mRNA expression was an independent marker of prognosis in stage II and stage III colorectal cancer. For *NXPE1*, we found two studies, which were bioinformatics studies, but they did not determine the function of this gene. Although there are few studies on the functions of these genes, we used GO analysis and KEGG pathway analysis to predict the possible function of the genes and the possible signaling pathways, providing a direction for subsequent functional research.

The expression pattern of these seven DEGs was confirmed by 672 RNA‐seq data on TCGA. Their expression levels presented the same trend as that in the GEO database, except for *MUC12*. However, *MUC12* was also downregulated in colorectal cancer tissues compared with normal colorectal mucosa, and its expression levels in our own patient validation cases were consistent with the predictions of the GEO database, indicating that *MUC12* is a promising marker. However, one‐third of cases in TCGA were extracted again for further prognosis validation, and all seven DEGs showed a close relationship with patient prognosis (Table S4).

We also confirmed the expression levels and the relationship with prognosis of these seven DEGs using patients in our hospital for medical treatment. Gene sequencing was performed on the surgical samples of 28 patients with CRC who had not received antitumor treatment. Consistent with the bioinformatics predictions, *MUC12* and *NXPE1* presented a sequentially descending trend in expression with tumor progression, and *TIMP1* presented a sequentially ascending trend (Table 5). Among these genes, only *NXPE1* was considered related to patient prognosis of the three‐year survival rate (Fig. S3, Table 7). There was a much larger mismatch between the validation results of our own patient cases and TCGA RNA‐seq data, probably because of the scarcity of patients compared with the large number of validation cases in TCGA. Nevertheless, our findings suggest that DEGs play an important role in the development of CRC.

**Table 7:** The expression level and the relationship with prognosis of seven DEGs from patients in our hospital for medical treatment

Genes	Normal stage 1 |FC|	Normal stage 2 |FC|	Normal stage 3 |FC|	Normal stage 4 |FC|	Three‐year survival rate (*P*‐value)
GCG	−5.32297	−3.3835	−4.15322	−5.56661	0.95118
HEPACAM2	−3.09828	−1.72218	−2.58992	−1.76615	0.09793
ITLN1	−1.86113	−1.51937	−1.64402	−1.13096	0.8532
LGALS2	−3.18707	−2.26365	−2.63189	−2.53429	0.0765
MUC12	−1.37528	−2.07949	−2.08844	−2.87224	0.14525
NXPE1	−2.06956	−2.71137	−2.60975	−2.77593	0.0068
TIMP1	1.00443	2.180932	2.418588	2.083604	0.2753

DEGs, differentially expressed genes; FC, fold change.

There are several limitations in our study. First, the amount of data we obtained from the GEO database and our validation were still not sufficient. However, these were all the data we could obtain from the GEO while still insuring data quality. In the future, more qualified patients in our hospital for medical treatment should be followed up. Second, the data in the GEO database are based on different experimental studies, and there was a lack of uniform standards, which would add heterogeneity to our findings. Thus, we attempted to minimize heterogeneity and to insure the rigor of the data by only including GPL570 platform data, performing quality assessment of the microarray and preprocessing the data. Although the batch effect was evaluated across the different datasets, and the heterogeneity was not significant, it still exists.

In conclusion, we discovered seven DEGs through the normal colorectal mucosa–adenoma–carcinoma sequence associated with CRC patient prognosis. Six genes that present sequential expression level changes with different stages might reflect the degree of tumor progression. One downregulated gene might play a key role in the early stages of neoplasia. Monitoring changes in these gene expression levels will allow us to assess disease progression, evaluate treatment efficacy, and predict prognosis. In addition, our study provides a set of useful targets for further functional research exploring the molecular mechanisms and uncovering new therapeutic targets.

## Ethical Approval

All procedures performed in studies involving human participants were in accordance with the ethical standards of the institutional and/or national research committee and with the 1964 Helsinki declaration and its later amendments or comparable ethical standards. For this type of study, formal consent is not required.

## Conflict of Interest

The authors declare that they have no conflict of interest.

## Supporting information


**Figure S1.** Quality assessment.

**Figure S2.** Batch effect was evaluated with the expression level of GAPDH across the different datasets, and heterogeneity was not significant (*P* > 0.05).

**Figure S3.** Survival curve of **A.** HEPACAM2, **B.** ITLN1, **C.** LGALS2, **D.** MUC12, **E.** NXPE1, **F.** TIMP1 and **G.** GCG from patients in our hospital for medical treatment.

**Table S1.** Search strategies.
**Table S2.** Summary of thirty‐eight datasets.
**Table S3.** Eighty‐seven genes present sequentially expression level changes through normal colorectal mucosa‐adenoma‐carcinoma sequence.
**Table S4.** Further prognosis validation with one‐third cases on TCGA.
