# Supplementary figures and images for: Analysis of potential genes and pathways associated with the colorectal normal mucosa–adenoma–carcinoma sequence
Source: Cancer Med. 2018 Apr 16;7(6):2555–66. doi: 10.1002/cam4.1484 (PMC6010713; doi:10.1002/cam4.1484)

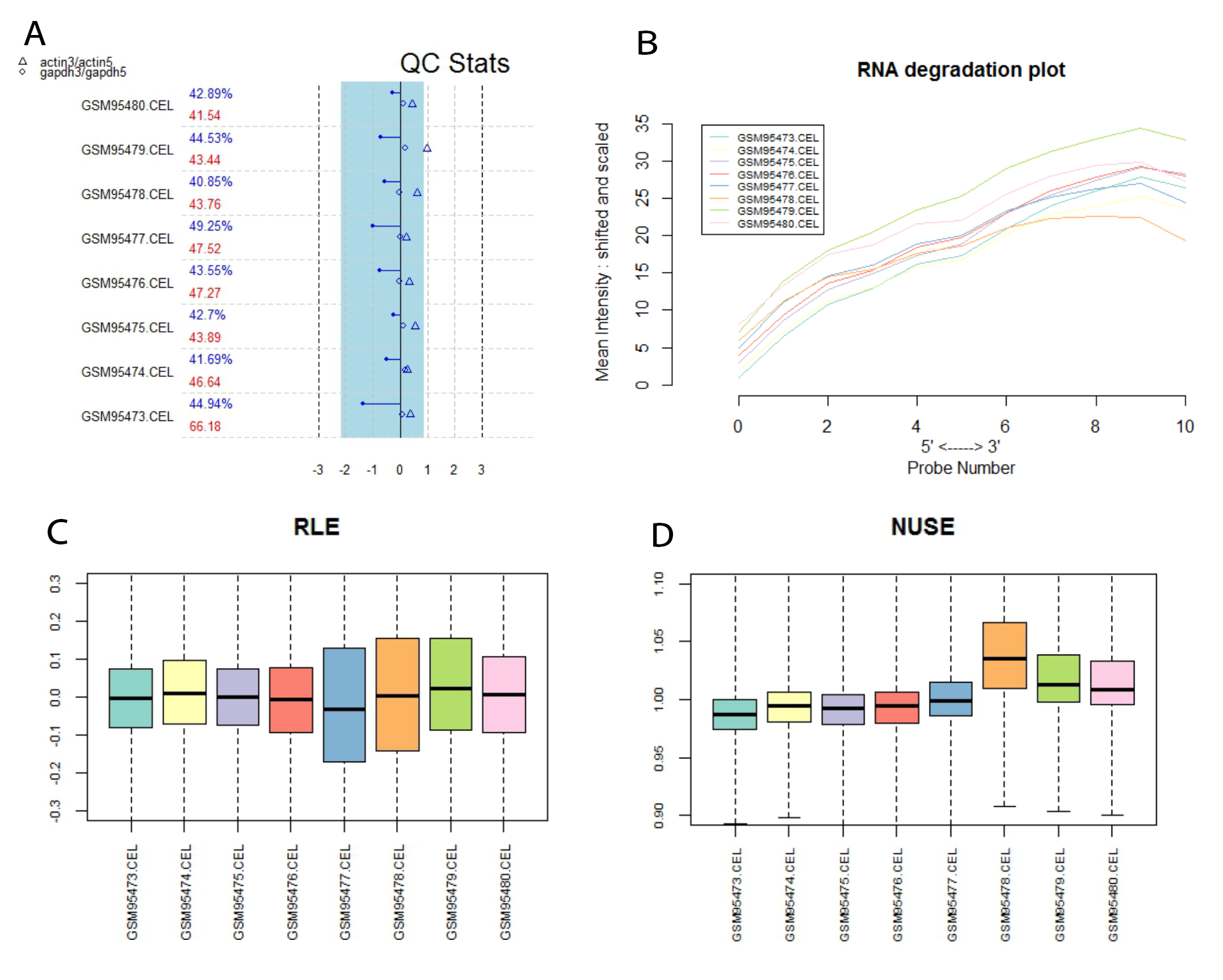

Supplement: Supplementary file 1 — Figure S1. Quality assessment. [file CAM4-7-2555-s001.tif]

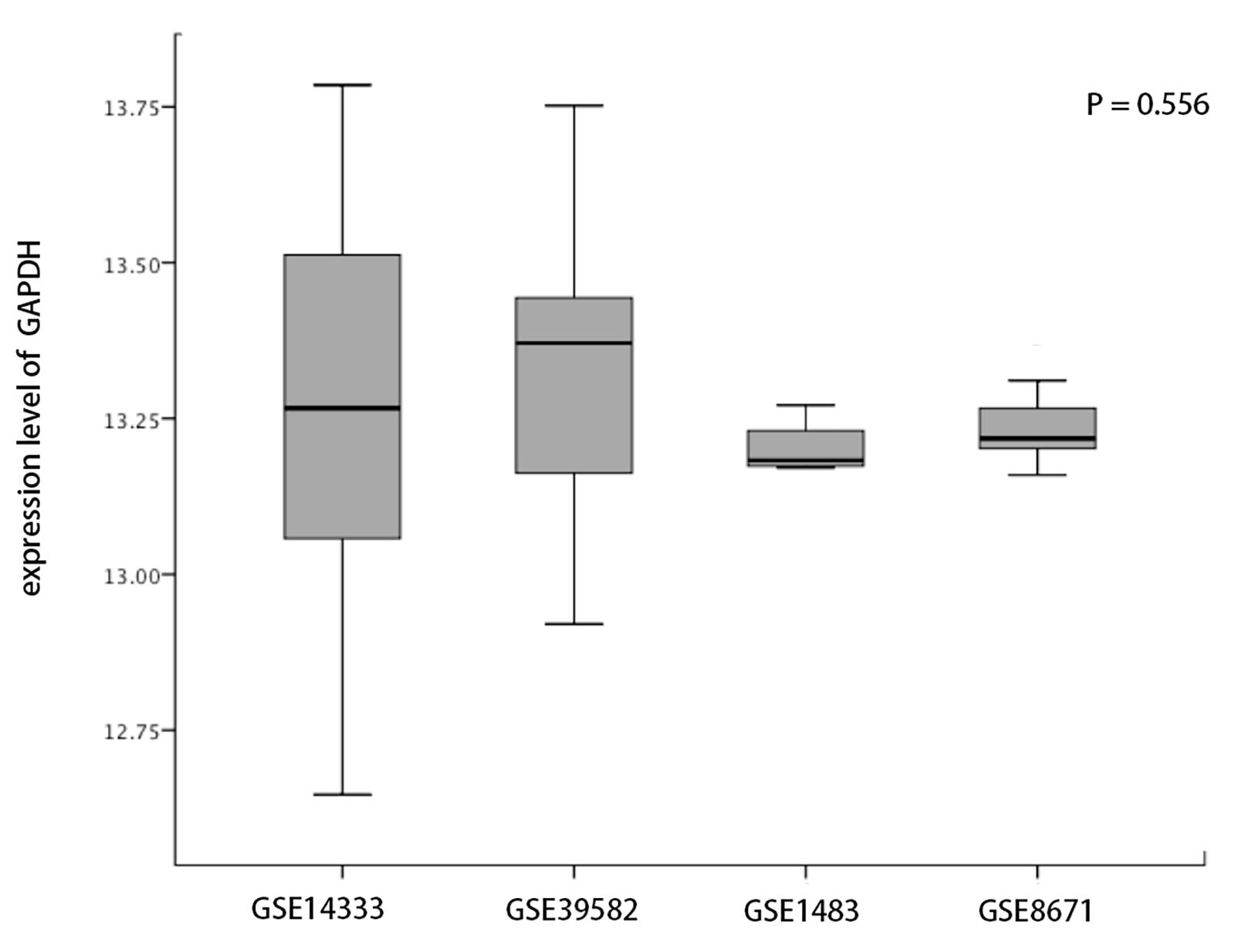

Supplement: Supplementary file 2 — Figure S2. Batch effect was evaluated with the expression level of GAPDH across the different datasets, and heterogeneity was not significant (P > 0.05). [file CAM4-7-2555-s002.tif]

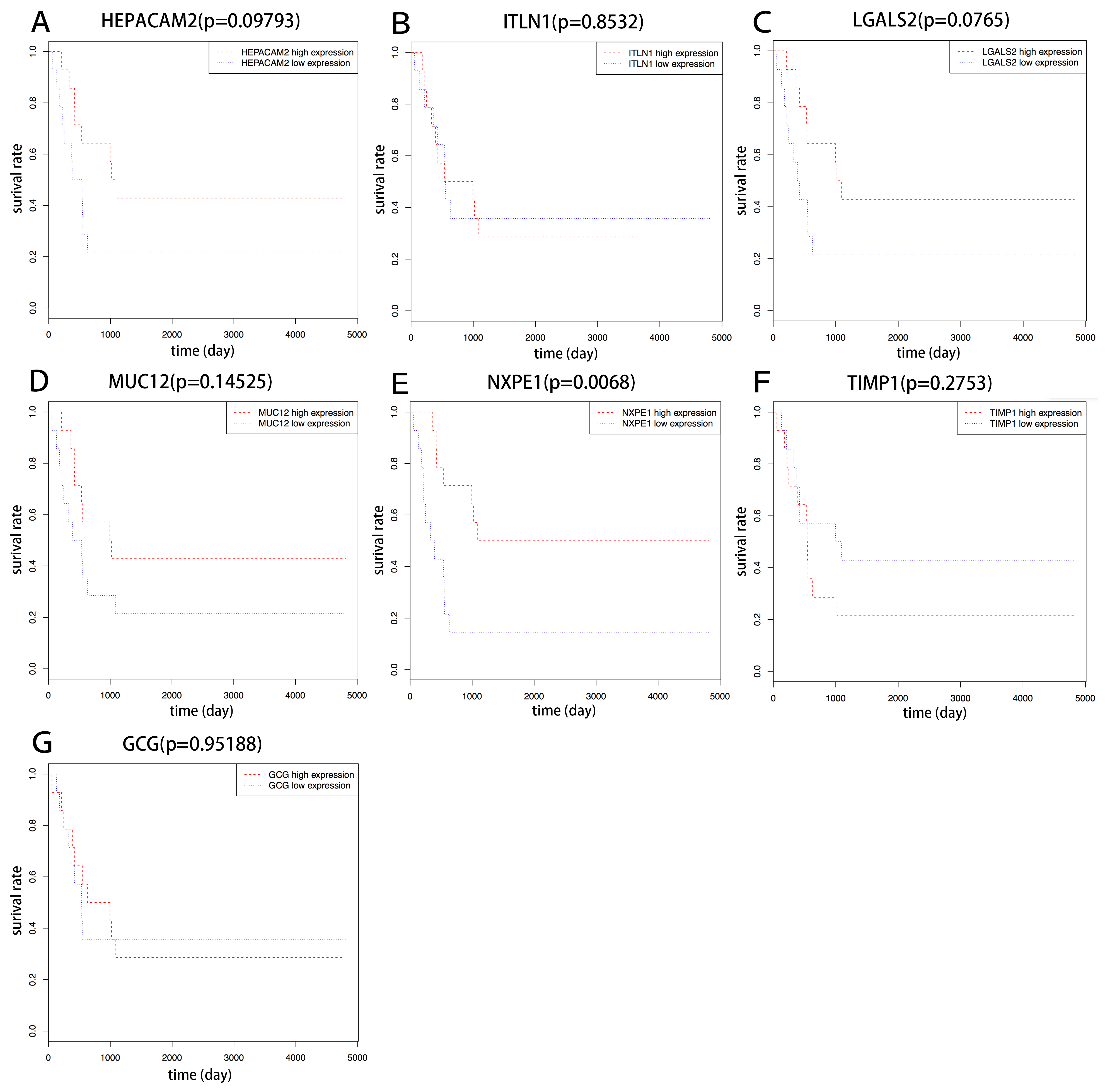

Supplement: Supplementary file 3 — Figure S3. Survival curve of A. HEPACAM2, B. ITLN1, C. LGALS2, D. MUC12, E. NXPE1, F. TIMP1 and G. GCG from patients in our hospital for medical treatment. [file CAM4-7-2555-s003.tif]
