# Supplementary material for: Analysis of potential genes and pathways associated with the colorectal normal mucosa–adenoma–carcinoma sequence
Source: Cancer Med. 2018 Apr 16;7(6):2555–66. doi: 10.1002/cam4.1484 (PMC6010713; doi:10.1002/cam4.1484)
Supplement: Supplementary file 4 — Table S1. Search strategies. Table S2. Summary of thirty‐eight datasets. Table S3. Eighty‐seven genes present sequentially expression level changes through normal colorectal mucosa‐adenoma‐carcinoma sequence. Table S4. Further prognosis validation with one‐third cases on TCGA. [file CAM4-7-2555-s004.docx]

**Supplementary table 1. Search strategies**

| No | Searches | Results |
| --- | --- | --- |
| 1 | Search "Colorectal Neoplasms"[MeSH Terms] | 19866 |
| 2 | Search ((((colon*[Description]) OR rectal*[Description]) OR colorect*[Description])) AND (((((tumo*[Description]) OR cancer*[Description]) OR carcinom*[Description]) OR neoplas*[Description]) OR adenocarcinoma*[Description]) | 33989 |
| 3 | 1 OR 2 | 36752 |
| 4 | Search ((profil*[Description]) OR pattern*[Description]) OR expression[Description] | 390122 |
| 5 | Search mRNA[Description] | 35359 |
| 6 | 3 AND 4 AND 5 | 621 |
| 7 | "Homo sapiens"[porgn] | 1225777 |
| 8 | 6 AND 7 | 592 |

NOTE: Searched up to January 7, 2017 at GEO database.

**Supplementary table 2. Summary of thirty-eight datasets**

| GEO series | Platform | Staging information (yes/no) | Receiving anti-tumor treatments shortly before (yes/no) |
| --- | --- | --- | --- |
| GSE4183 | GPL570 | Yes | No |
| GSE14333 | GPL570 | Yes | No |
| GSE39582 | GPL570 | Yes | No |
| GSE8671 | GPL570 | Yes | No |
| GSE10714 | GPL570 | Yes | No |
| GSE60697 | GPL570 | Yes | Yes |
| GSE20916 | GPL570 | Yes | NR |
| GSE21510 | GPL570 | Yes | NR |
| GSE22242 | GPL570 | Yes | NR |
| GSE27854 | GPL570 | Yes | NR |
| GSE29621 | GPL570 | Yes | NR |
| GSE32323 | GPL570 | Yes | NR |
| GSE37364 | GPL570 | Yes | NR |
| GSE38832 | GPL570 | Yes | NR |
| GSE45270 | GPL570 | Yes | NR |
| GSE33113 | GPL570 | Yes | NR |
| GSE17538 | GPL570 | Yes | NR |
| GSE75316 | GPL570 | Yes | NR |
| GSE22598 | GPL570 | No | / |
| GSE79462 | GPL13158 | / | / |
| GSE79460 | GPL13158 | / | / |
| GSE75050 | GPL16956 | / | / |
| GSE63596 | GPL17077 | / | / |
| GSE79038 | GPL16686 | / | / |
| GSE41657 | GPL6480 | / | / |
| GSE62321 | GPL97 | / | / |
| GSE35834 | GPL15236 | / | / |
| GSE49355 | GPL92 | / | / |
| GSE40966 | GPL16070 | / | / |
| GSE35982 | GPL4133 | / | / |
| GSE20970 | GPL1528 | / | / |
| GSE12225 | GPL2641 | / | / |
| GSE77953 | GPL96 | / | / |
| GSE76713 | GPL16228 | / | / |
| GSE41657 | GPL6480 | / | / |
| GSE47063 | GPL6102 | / | / |
| GSE57965 | GPL15207 | / | / |
| GSE29915 | GPL13716 | / | / |

NOTE, NR: Not reported, GEO: Gene Expression Omnibus, GSE: GEO series, GPL: GEO platform.

**Supplementary table 3. Eighty-seven genes present sequentially expression level changes through normal colorectal mucosa-adenoma-carcinoma sequence**

| Genes | Normal-  adenoma  \|FC\| | Normal-  stage 1  \|FC\| | Normal-  stage 2  \|FC\| | Normal-  stage 3  \|FC\| | Normal-  stage 4  \|FC\| | Five-year survival rate (P-value) |
| --- | --- | --- | --- | --- | --- | --- |
| ABCA8 | -1.41970 | -1.38258 | -1.35370 | -1.31892 | -1.28234 | 0.55596 |
| ADH1C | -2.31924 | -3.53965 | -4.10021 | -3.83618 | -4.17274 | 0.79018 |
| AGPAT9 | -1.47403 | -2.32395 | -2.59011 | -2.34702 | -2.59879 | NA |
| AHCYL2 | -1.11236 | -1.80287 | -2.00768 | -1.77677 | -2.21828 | 0.37324 |
| AKR1B10 | -1.00410 | -3.25147 | -3.51545 | -3.26215 | -3.79440 | 0.25223 |
| AQP8 | -5.08128 | -5.59115 | -5.85151 | -5.60485 | -6.51036 | 0.13895 |
| BEST2 | -1.90373 | -3.32708 | -3.15763 | -3.24307 | -3.66477 | 0.35462 |
| C2CD4A | 3.91608 | 3.45816 | 2.64656 | 2.52755 | 2.64169 | 0.20969 |
| C2orf88 | -2.06778 | -3.02886 | -3.05864 | -2.97201 | -3.47512 | 0.23554 |
| CA2 | -2.67355 | -3.79929 | -4.71954 | -4.04259 | -4.84264 | 0.07362 |
| CA4 | -3.90284 | -4.12978 | -4.42648 | -4.23627 | -4.70592 | 0.17506 |
| CDHR5 | -1.33046 | -2.30253 | -2.39422 | -2.32497 | -2.53937 | 0.69486 |
| CEACAM7 | -2.56245 | -3.76971 | -4.58976 | -3.91128 | -4.87991 | 0.21383 |
| CHP2 | -2.34803 | -3.08140 | -3.39057 | -3.36710 | -3.77139 | 0.59621 |
| CLCA4 | -4.01680 | -5.75406 | -5.75458 | -5.46981 | -6.55938 | 0.16333 |
| CLDN8 | -4.71209 | -5.19946 | -5.58836 | -5.06006 | -5.65663 | 0.9914 |
| CNTN3 | -1.48060 | -2.42554 | -3.07506 | -2.85534 | -2.60021 | 0.17508 |
| CWH43 | -2.19901 | -3.01561 | -3.86950 | -3.92771 | -3.70659 | 0.63255 |
| CXCL8 | 2.83059 | 3.49790 | 4.03893 | 4.29831 | 3.54721 | 0.11662 |
| DHRS11 | -2.00594 | -2.54025 | -2.68959 | -2.81866 | -2.95578 | 0.14795 |
| DHRS9 | -1.61759 | -3.19316 | -3.19429 | -2.59701 | -3.37786 | 0.68233 |
| DUSP27 | 3.37896 | 1.66721 | 1.51201 | 1.47605 | 1.60661 | 0.84341 |
| ENTPD5 | -1.11896 | -1.99494 | -2.03975 | -2.04521 | -2.13646 | 0.75755 |
| FLJ22763 | -1.26056 | -1.70686 | -1.94764 | -1.92928 | -2.10480 | 0.4012 |
| GALNT12 | -1.22250 | -1.40332 | -1.63121 | -1.69906 | -1.94731 | 0.26501 |
| GDPD3 | -1.07776 | -1.59181 | -1.71245 | -1.72676 | -1.79987 | 0.28652 |
| GREM1 | -1.13405 | 1.28095 | 2.30494 | 2.48717 | 2.36991 | 0.27448 |
| HEPACAM2 | -1.21761 | -3.85613 | -4.07060 | -4.06757 | -4.57233 | 0.00029 |
| HRCT1 | -1.28402 | -1.91091 | -2.39116 | -2.53764 | -2.22235 | 0.66674 |
| HSD11B2 | -1.01858 | -2.27925 | -2.88567 | -2.87908 | -2.82204 | 0.57168 |
| HSD17B2 | -2.17929 | -3.77401 | -3.94716 | -3.58411 | -4.13218 | 0.45062 |
| IL1R2 | -1.75110 | -2.93584 | -2.96938 | -2.65013 | -3.30852 | 0.48492 |
| ISX | -1.45893 | -2.11180 | -2.64186 | -2.78839 | -2.43025 | 0.27509 |
| ITLN1 | -2.18651 | -4.00026 | -4.54605 | -4.00429 | -4.82109 | 0.00215 |
| KRT20 | -1.10093 | -2.07680 | -2.18192 | -1.80302 | -2.49438 | 0.74703 |
| LAMA1 | -1.03341 | -1.88857 | -1.94457 | -1.94811 | -2.15253 | 0.90957 |
| LGALS2 | -1.86415 | -2.78240 | -3.50298 | -3.01946 | -3.08688 | 0.04796 |
| LINC00483 | -1.05606 | -1.91321 | -2.28445 | -2.25558 | -2.33659 | 0.67279 |
| LOC100293211 | -1.05262 | -1.51343 | -2.05447 | -2.08252 | -1.98635 | NA |
| LRRC19 | -1.50095 | -2.47273 | -2.94515 | -3.02241 | -3.29629 | 0.59757 |
| MACC1 | 1.04198 | 1.63846 | 1.84498 | 1.98778 | 1.81049 | 0.8463 |
| MALL | -1.60786 | -2.07238 | -2.31970 | -2.22764 | -2.51207 | 0.21534 |
| MEP1A | -1.83633 | -2.08504 | -2.48864 | -2.79149 | -2.64534 | 0.44236 |
| MFSD4 | -1.20247 | -1.84526 | -1.98190 | -1.85330 | -2.13576 | NA |
| MS4A12 | -4.48105 | -5.56404 | -5.52912 | -5.06747 | -6.16997 | 0.74113 |
| MT1M | -2.44922 | -4.44343 | -4.18248 | -3.74409 | -4.39781 | 0.28176 |
| MUC12 | -1.08214 | -2.12846 | -2.39063 | -2.56680 | -2.54186 | 0.03171 |
| NR3C2 | -1.22246 | -2.13283 | -2.85683 | -2.83000 | -2.86369 | 0.45219 |
| NR5A2 | -1.68519 | -2.03326 | -2.29748 | -2.16524 | -2.25627 | 0.23317 |
| NXPE1 | -1.00081 | -2.57598 | -2.93155 | -2.77359 | -3.22360 | 0.03647 |
| NXPE4 | -1.47968 | -3.14661 | -3.45173 | -3.23103 | -3.62841 | 0.17931 |
| PADI2 | -1.15365 | -1.53613 | -1.82114 | -1.76890 | -1.93479 | 0.72186 |
| PAQR5 | -1.23598 | -1.24246 | -1.54911 | -1.66454 | -1.64907 | 0.93564 |
| PIGZ | -1.17587 | -1.94427 | -2.17059 | -2.50482 | -1.90519 | 0.68269 |
| PKIB | -2.78018 | -3.34695 | -3.81747 | -3.49068 | -3.98953 | 0.28921 |
| PLAC8 | -1.33451 | -2.88712 | -2.80513 | -2.29729 | -3.20461 | 0.68558 |
| PNLIPRP2 | -1.97703 | -2.37260 | -2.56758 | -2.74392 | -2.44758 | 0.16225 |
| PPBP | 1.01794 | 1.06278 | 2.07252 | 2.26301 | 2.01460 | 0.60527 |
| QPCT | 2.12463 | 1.04004 | 1.08654 | 1.02562 | 1.06703 | 0.74339 |
| REG1B | 3.35693 | 2.00020 | 2.00473 | 1.72894 | 1.83953 | 0.50369 |
| S100P | 2.62367 | 1.09809 | 1.33650 | 1.44361 | 1.23463 | 0.87716 |
| SCGB2A1 | -1.42922 | -3.23287 | -3.58812 | -2.98734 | -3.65299 | 0.09295 |
| SEMA6A | -1.09940 | -1.67712 | -1.88344 | -1.91043 | -1.80336 | 0.29419 |
| SI | -3.00848 | -4.27851 | -4.48132 | -3.47494 | -4.46304 | 0.84433 |
| SLAMF7 | -1.04954 | -1.33103 | -1.44935 | -1.37288 | -1.66204 | 0.05736 |
| SLC16A9 | -1.43962 | -2.28882 | -3.08982 | -2.92117 | -3.06016 | 0.54541 |
| SLC1A1 | -1.31902 | -1.50299 | -1.69202 | -1.56540 | -1.90970 | 0.30715 |
| SLC22A18AS | -1.15934 | -1.20239 | -1.47038 | -1.47767 | -1.57508 | 0.61096 |
| SLC26A2 | -3.05807 | -3.41037 | -3.77960 | -3.72670 | -4.09442 | 0.82135 |
| SLC4A4 | -2.66824 | -3.08682 | -3.30276 | -2.98008 | -3.62440 | 0.29116 |
| SLC6A14 | 2.41188 | 1.67755 | 1.37781 | 1.79936 | 1.25138 | 0.86808 |
| SRI | -1.03470 | -1.25858 | -1.50471 | -1.35272 | -1.57262 | 0.8403 |
| STAP2 | -1.01567 | -1.52810 | -1.68305 | -1.75023 | -1.64886 | 0.15135 |
| TCN1 | 4.77987 | 2.21081 | 2.13383 | 2.24126 | 1.71125 | 0.79071 |
| TEX11 | -1.42911 | -1.67209 | -1.90941 | -1.90205 | -1.90509 | 0.0588 |
| TIMP1 | 1.56175 | 1.69032 | 2.03628 | 2.05078 | 2.21735 | 0.0025 |
| TMCC3 | -1.58106 | -1.89226 | -2.06862 | -1.64975 | -2.18873 | NA |
| TMEM171 | -1.18041 | -1.52092 | -1.65156 | -1.60344 | -1.89312 | 0.93391 |
| TMEM220 | -1.27356 | -1.73963 | -1.92409 | -1.93006 | -1.90900 | 0.26227 |
| TNFRSF17 | -2.15014 | -2.68806 | -3.80498 | -3.60940 | -3.48755 | 0.10645 |
| TRANK1 | -1.04787 | -1.18636 | -1.43525 | -1.45719 | -1.16882 | 0.87346 |
| TSPAN7 | -2.13039 | -2.50398 | -2.58320 | -2.55699 | -2.68621 | 0.79741 |
| TUBAL3 | -1.48168 | -2.46815 | -2.84596 | -3.03424 | -2.87670 | 0.13762 |
| UGT2A3 | -2.43708 | -2.90010 | -3.23381 | -2.91703 | -3.86264 | 0.18867 |
| UGT2B17 | -3.43610 | -4.67742 | -4.71893 | -4.89797 | -5.62691 | 0.55315 |
| VIPR1 | -1.15556 | -1.58845 | -2.05139 | -2.10362 | -1.85569 | 0.24237 |
| ZG16 | -3.24504 | -5.20446 | -5.57551 | -5.24996 | -5.75655 | 0.17071 |

NOTE, NA: Not available, FC: Fold change.

**Supplementary table 4. Further prognosis validation with one-third cases on TCGA**

| Genes | Five-year survival rate (P-value) |
| --- | --- |
| HEPACAM2 | 0.0001 |
| ITLN1 | 0.0005 |
| LGALS2 | 0.0445 |
| MUC12 | 0.0451 |
| NXPE1 | 0.0159 |
| TIMP1 | 0.0006 |
| GCG | 0.0192 |
