# Supplementary material for: Analysis of potential genes and pathways associated with the colorectal normal mucosa–adenoma–carcinoma sequence
Source: Cancer Med. 2018 Apr 16;7(6):2555–66. doi: 10.1002/cam4.1484 (PMC6010713; doi:10.1002/cam4.1484)
Supplement: Supplementary file 5 [file CAM4-7-2555-s005.docx]

**Supplementary Figure legends**

**Supplementary figure 1. Quality assessment.** Normal colorectal mucosa arrays of GSE4183 were evaluated for quality by **A.** quality control (QC), **B.** RNA degradation curve, **C.** relative logarithmic expression (RLE) and **D.** normalized unscaled standard errors (NUSE).

**Supplementary figure 2.** Batch effect was evaluated with the expression level of GAPDH across the different datasets, and heterogeneity was not significant (p > 0.05).

**Supplementary figure 3.** Survival curve of **A.** HEPACAM2, **B.** ITLN1, **C.** LGALS2, **D.** MUC12, **E.** NXPE1, **F.** TIMP1 and **G.** GCG from patients in our hospital for medical treatment.
